# Phenotypic alteration of CD8^+^ T cells in chronic lymphocytic leukemia is associated with epigenetic reprogramming

**DOI:** 10.18632/oncotarget.9941

**Published:** 2016-06-10

**Authors:** Jiazhu Wu, Xiaojing Xu, Eun-Joon Lee, Austin Y. Shull, Lirong Pei, Farrukh Awan, Xiaoling Wang, Jeong-Hyeon Choi, Libin Deng, Hong-Bo Xin, Wenxun Zhong, Jinhua Liang, Yi Miao, Yujie Wu, Lei Fan, Jianyong Li, Wei Xu, Huidong Shi

**Affiliations:** ^1^ Department of Hematology, the First Affiliated Hospital of Nanjing Medical University, Jiangsu Province Hospital, Nanjing, Jiangsu, 210029, China; ^2^ Collaborative Innovation Center for Cancer Personalized Medicine, Nanjing Medical University, Nanjing, Jiangsu, 210029, China; ^3^ Georgia Cancer Center, Augusta University, Augusta, GA 30912, USA; ^4^ Georgia Prevention Institute, Augusta University, Augusta, GA 30912, USA; ^5^ Department of Biochemistry & Molecular Biology, Medical College of Georgia, Augusta University, Augusta, GA 30912, USA; ^6^ Division of Hematology, The Ohio State University, Columbus, OH, 43210, USA; ^7^ Institute of Translational Medicine, Nanchang University, Nanchang 330031, China; ^8^ Department of Statistics, University of Georgia, Athens, GA 30602, USA

**Keywords:** chronic lymphocytic leukemia, DNA methylation, CD8^+^ T-cells, PD-1

## Abstract

Immunosuppression is a prevalent clinical feature in chronic lymphocytic leukemia (CLL) patients, with many patients demonstrating increased susceptibility to infections as well as increased failure of an antitumor immune response. However, much is currently not understood regarding the precise mechanisms that attribute to this immunosuppressive phenotype in CLL. To provide further clarity to this particular phenomenon, we analyzed the T-cell profile of CLL patient samples within a large cohort and observed that patients with an inverted CD4/CD8 ratio had a shorter time to first treatment as well as overall survival. These observations coincided with higher expression of the immune checkpoint receptor PD-1 in CLL patient CD8^+^ T cells when compared to age-matched healthy donors. Interestingly, we discovered that increased PD-1 expression in CD8^+^ T cells corresponds with decreased DNA methylation levels in a distal upstream locus of the PD-1 gene *PDCD1*. Further analysis using luciferase reporter assays suggests that the identified *PDCD1* distal upstream region acts as an enhancer for *PDCD1* transcription and this region becomes demethylated during activation of naïve CD8^+^ T cells by anti-CD3/anti-CD28 antibodies and IL2. Finally, we conducted a genome-wide DNA methylation analysis comparing CD8^+^ T cells from CLL patients against healthy donors and identified additional differentially methylated genes with known immune regulatory functions including *CCR6* and *KLRG1*. Taken together, our findings reveal the occurrence of epigenetic reprogramming taking place within CLL patient CD8^+^ T cells and highlight the potential mechanism of how immunosuppression is accomplished in CLL.

## INTRODUCTION

Chronic lymphocytic leukemia (CLL), the most common type of adult leukemia in the western world, is characterized by the progressive accumulation of monoclonal B cells. The clinical progression among CLL patients can be highly heterogeneous as some patients experience rapid disease progression while others can live for decades without ever requiring treatment [1, 2]. CLL patients also can be presented with immune deficiency issues that increase with disease progression, thus resulting in failure of antitumor immunity as well as increased susceptibility to infections. Previous studies have revealed global alterations in the gene expression profiles of T cells from patients with CLL compared to age-matched healthy controls [3]. It also has been documented that CLL cells can induce profound defects in T-cell functions [3–7]. Specifically, CLL cells are shown to express multiple inhibitory ligands such as CD200, CD274 (PD-L1), and CD276 (B7-H3), which can cause impaired actin synapse formation in allogeneic and autologous T cells [5]. Furthermore, T cells from the Eμ-TCL1 transgenic CLL mouse model exhibit similar gene expression profiles and functional defects as those seen in CLL patients [8, 9]. The abnormal T-cell compartment is also a part of the supporting milieu, which sustains and prevents apoptosis of CLL cells *in vivo* and *in vitro* [7, 10].

T-cell exhaustion, which is defined as a state of T-cell dysfunction that can arise during both chronic viral infection and cancer development, has been identified in CLL [11]. Exhausted T cells are generally associated with poor effector function, loss of proliferative capacity, impaired cytotoxicity, and reduced cytokine production. CD8^+^ T cells from CLL patients exhibit increased expression of inhibitory receptors that correspond with the T-cell exhaustion phenotype in chronic infections including programmed death 1 (PD-1, CD279), CD244, and CD160 [11, 12]. Recent studies suggest that PD-L1 checkpoint blockade prevents immune dysfunction and leukemia development in the Eμ-TCL1 transgenic CLL mouse model [13, 14]. Therefore, targeting the PD-1/PD-L1 axis has been suggested as a therapeutic approach that should be further explored in clinical studies with CLL patients, ideally in combination with novel compounds to help eliminate CLL cells [14].

Though phenotypic alterations of CLL T cells have been reported, the molecular mechanism driving T-cell dysfunction in CLL remains poorly understood. Mounting evidence suggests that epigenetic regulation plays an important role in the differentiation of T cells and may serve as a mechanism to preserve ‘poised’ transcription states in antigen-specific T cells [15]. The most extensively studied epigenetic mark is DNA methylation, which can support long-term memory of altered functional properties [15, 16]. A previous study demonstrated that mouse and human antigen-specific CD8^+^ T cells that undergo virus-induced differentiation express high levels of PD-1 [17]. Interestingly, the study also demonstrated that PD-1 up-regulation coincided with demethylation of the PD-1 *cis-*regulatory region, and this demethylated state was maintained as a result of chronic antigen stimulation [17]. Based on these observations, we hypothesize that similar epigenetic alterations are also involved in T-cell dysfunction within CLL patients. To test our hypothesis, we examined the methylation status of the PD-1 locus in CD8^+^ T cells from CLL patients and demonstrated the role of DNA methylation in regulating PD-1 expression. We further conducted a genome-wide DNA methylation analysis in CD8^+^ T cells from CLL and healthy control samples and identified other differentially methylated genes with known functions in the immune system. Our results suggest that the exhaustion phenotype observed in CLL CD8^+^ T cells is associated with altered DNA methylation profiles in immune regulatory genes.

## RESULTS

### Inverted CD4/CD8 ratio is associated with poor outcome in CLL patients

**Figure 1:**
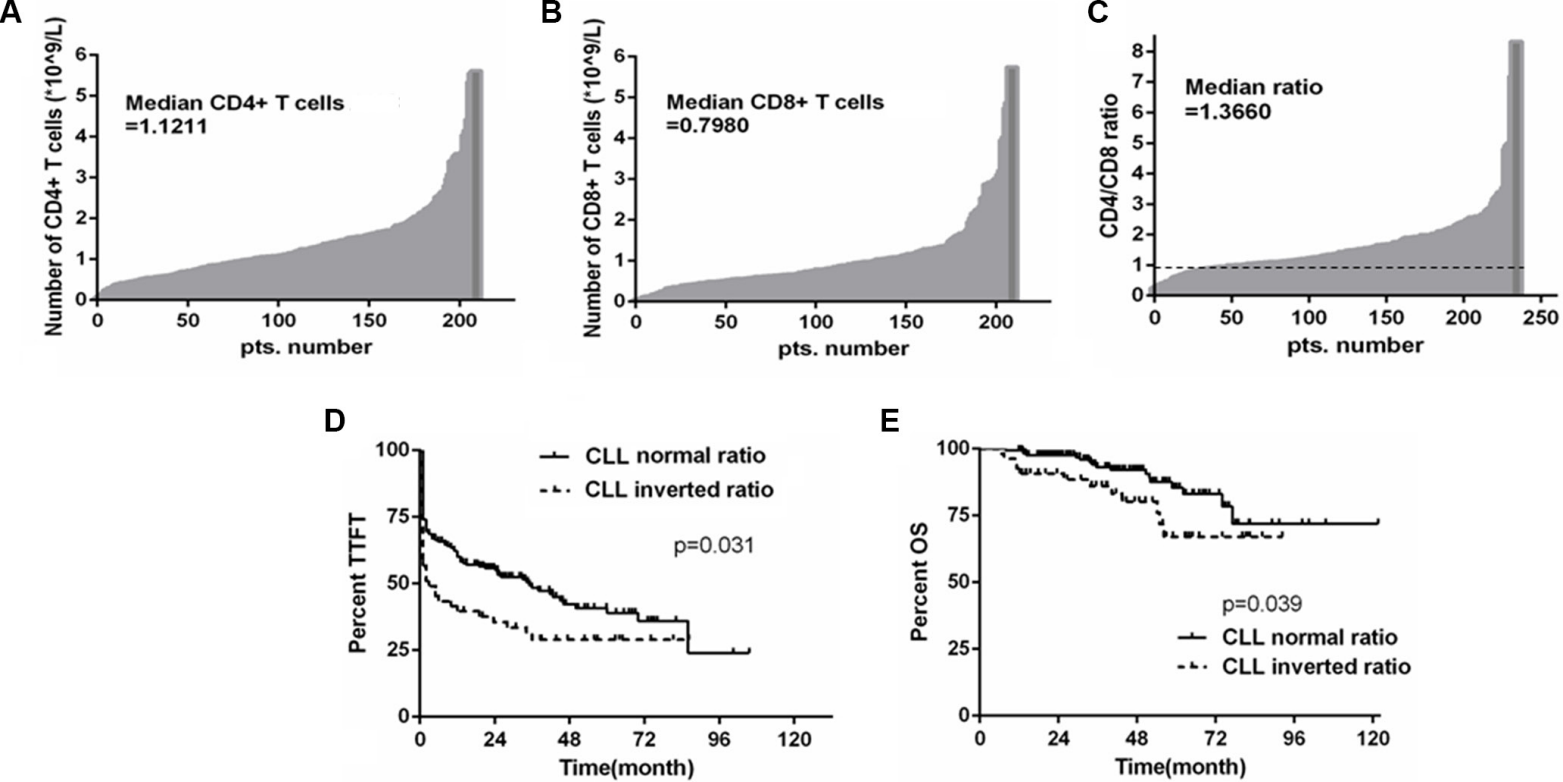
The inverted CD4/CD8 ratio is associated with poor outcome in CLL patients (**A**) and (**B**) Histograms illustrating the absolute numbers of CD4^+^ and CD8^+^ T cells in 234 Chinese CLL patients, respectively. (**C**) CD4/CD8 ratio was determined for the same group of CLL patients with the threshold between normal and inverted ratio being 1. (**D** and **E**) Kaplan-Meier survival analysis of TTFT and OS according to CD4/CD8 ratio; inverted ratio group referring to those below the cut-off value of 1, and normal ratio group as those above the cut-off value of 1 (*p* = 0.031 and *p* = 0.039, respectively).

It has been shown that the numbers of CD4^+^ and CD8^+^ T cells are associated with prognosis in CLL [18, 19]. In this study, we analyzed the absolute numbers of CD4^+^ and CD8^+^ T cells in the peripheral blood of CLL patients in a cohort of 234 Chinese CLL patients. The median number of CD8^+^ T cells in this cohort was 0.80 × 10^9^ cells/L, while the median number of CD4^+^ T cells was 1.12 × 10^9^ cells/L. 54 patients (23.1%) showed an inversion in the CD4/CD8 ratio based on a cutoff ratio of 1.0, indicating the CD8^+^ T-cell subset is larger than the CD4^+^ subset in this subgroup of CLL patients (Figures 1A–1C). We further examined whether any known prognostic markers are associated with the inverted CD4/CD8 ratio. A slightly significant association between inverted CD4/CD8 ratio and Binet stage (*p* = 0.039) was observed, whereas the comparison to CD38 expression fell just short of statistical significance (*p* = 0.054). However, no significant association with IGHV mutation status (*p* = 0.298), ZAP-70 expression (*p* = 0.098), or TP53 mutation or del(17 p) (*p* = 0.105) was observed (Table 1). Moreover, patients with the inverted CD4/CD8 ratio had shorter time to first treatment (TTFT) as well as shorter overall survival (OS) when compared to patients with normal CD4/CD8 ratio (*p* = 0.031 and *p* = 0.039, respectively) (Figures 1D–1E), a result consistent with previous studies of CLL patient cohorts [18, 19].

**Table 1:** Clinical characteristics of patients grouped by the CD4/CD8 ratio (cut off 1.0)

Patient characteristics	*n* = 234	CD4:CD8 ratio		
		< 1	> 1	*p*
Age (years)	61 (20–86)			
Gender (%)				
Male	159 (67.95%)			
Female	75 (32.05%)			
Binet stage (%)				
A/B	167 (71.37%)	32	135	0.039
C	67 (28.63%)	22	45	
IGHV (%) *n* = 197				
Mutated	122 (61.93%)	32	90	0.298
Unmutated	75 (38.07%)	14	61	
CD38 (%) *n* = 225				
Positive (> 30%)	48 (21.33%)	16	32	0.054
Negative (< 30%)	177 (78.67%)	35	142	
ZAP70 (%) *n* = 202				
Positive (> 20%)	88 (43.56%)	16	72	0.098
Negative (< 20%)	114 (56.44%)	33	81	
With TP53 mutation or del (17 p)	23 (13.69%)	8	15	0.105
Without TP53 mutation or del (17 p)	145 (86.31%)	28	117	

### PD-1 is upregulated in CD8^+^ T cells in CLL patients

**Figure 2:**
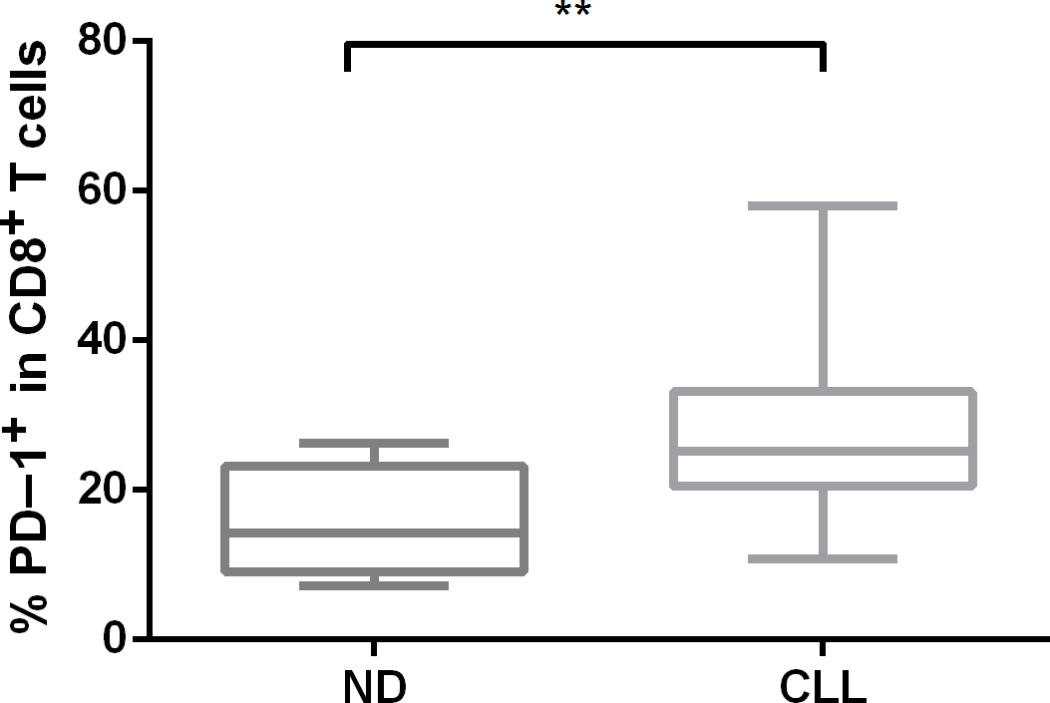
The surface expression of PD-1 in CD8^+^ T-cell subsets from CLL patients and normal donors PD-1 protein expression was measured by flow cytometry in primary CLL samples using an anti-PD-1 antibody.

The inverted CD4/CD8 ratio may be caused by preferential expansion of CD8^+^ terminal effector memory cells with a replicate senescence phenotype [19], so we analyzed the expression of PD-1 in our CLL patient cohort using flow cytometry. PD-1 is a marker of an “exhaustion” phenotype in CD8^+^ T cells and has been shown to be upregulated in CLL T cells [11, 20]. The percentage of PD-1^+^ cells was significantly higher in the CD8^+^ T-cell population of CLL patients (*n* = 22) when compared with normal age-matched controls (*n* = 10) (*p* = 0.001) (Figure 2).

### *PDCD1* upstream locus is hypomethylated in CD8^+^ T cells from CLL patients

**Figure 3:**
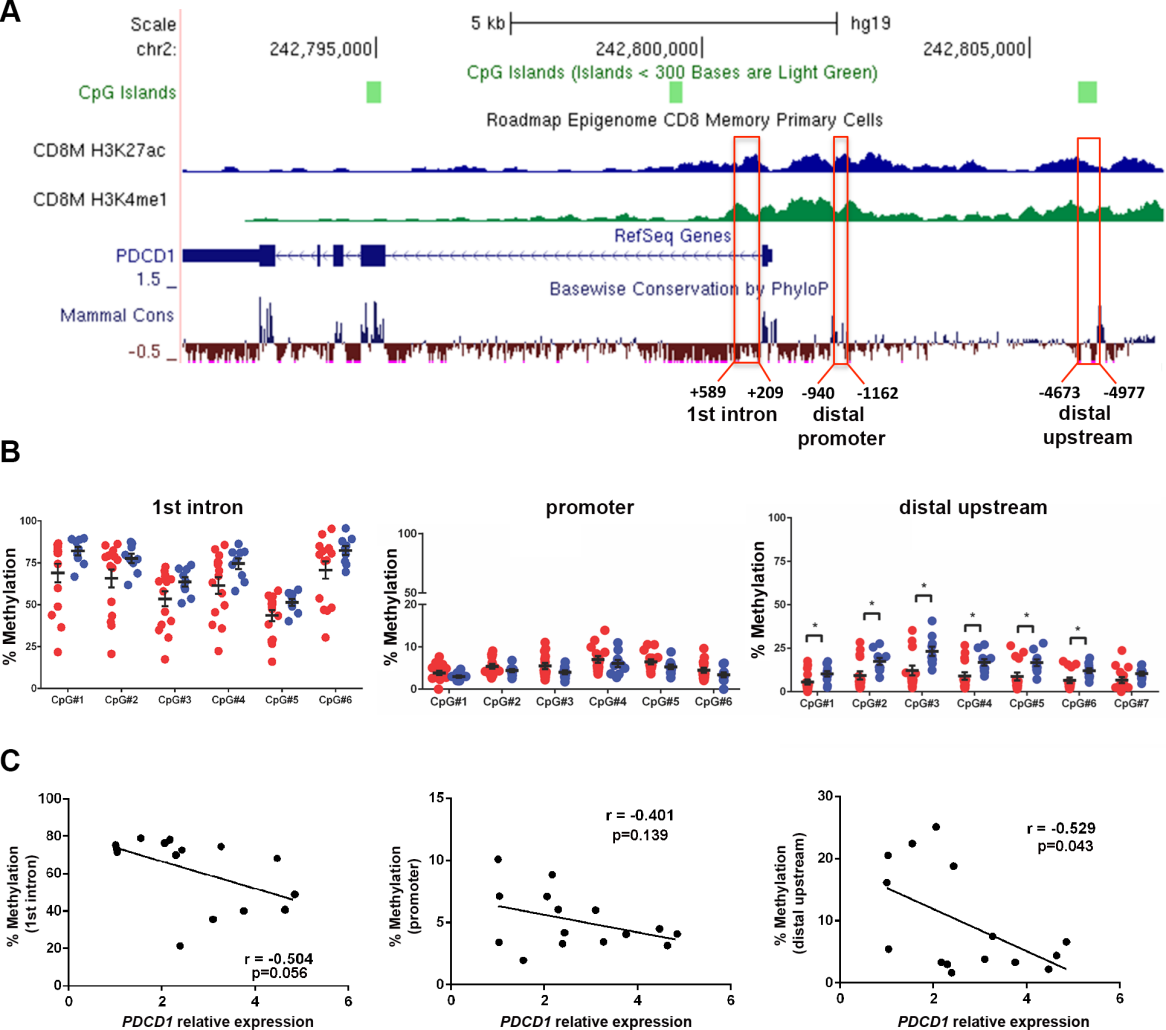
The distal upstream region of *PDCD1* is hypomethylated in CD8^+^ T cells from CLL patients (**A**) Illustration of the *PDCD1* gene from UCSC genome browser. The ChIP-seq tracks of H3K27ac and H3K4me1 marks in human CD8^+^ memory T cells were downloaded from the NIH Epigenome Roadmap dataset. Three candidate regulatory regions were selected for pyrosequencing analysis. (**B**) Bisulfite pyrosequencing results of first intron (+ 0.5 kb), promoter (−1 kb), and distal upstream (−4.7 kb) regions in CD8^+^ T cells from CLL and ND samples. Each dot represents a sample; red dots denote CD8^+^ T cells from CLL (*n* = 15) and blue dots represent ND samples (*n* = 10). *p < 0.05. (**C**) Correlation between the relative expression of *PDCD1* mRNA and the average methylation level of the first intron, promoter and distal upstream locus. Quantitative RT-PCR and bisulfite pyrosequencing analyses were performed on the matched CLL samples.

Previous reports show that chronic viral infection leads to demethylation of the regulatory regions of *PDCD1,* which encodes the PD-1 receptor, in mouse and human CD8^+^ T cells [17]. By investigating publically available human epigenome databases (http://genomebrowser.wustl.edu), we identified three candidate regulatory regions in the first intron (approximately +0.5 kb downstream), a promoter region (~ −1 kb), and a distal upstream region (~ −4.7 kp) from the transcription start sites (TSS), which are enriched in the enhancer-associated histone H3 lysine 27 acetylation (H3K27ac) and histone H3 lysine 4 monomethylation (H3K4me1) marks in human CD8^+^ memory T cells (Figure 3A). The distal promoter region (−1 kb) is referred as the conserved region C (CR-C) in a previous report [17]. We performed bisulfite pyrosequencing analysis in the three candidate regulatory regions in CD8^+^ T cells from 15 CLL and 10 normal donors (ND) samples. The quantitative pyrosequencing analysis demonstrated that the first intron region (+0.5 kb) was highly methylated, while the distal promoter region (−1 kb) was nearly unmethylated in CD8^+^ T cells from both CLL and ND samples. However, the −4.7 kb *PDCD1* upstream locus was significantly hypomethylated in CD8^+^ T cells from CLL patients as compared to ND samples (*p* < 0.05) (Figure 3B). When compared with qRT-PCR results of *PDCD1* mRNA in matched CLL samples, the average methylation levels of all three regions were inversely correlated with *PDCD1* mRNA levels, though only the −4.7 kb region reached statistical significance (*r* = −0.529, *p* = 0.043). These results suggest that methylation of *PDCD1*, particularly the −4.7 kb distal upstream region, may regulate *PDCD1* transcription in human CD8^+^ T cells (Figure 3C).

### CpG methylation regulates *PDCD1* upstream enhancer activity

**Figure 4:**
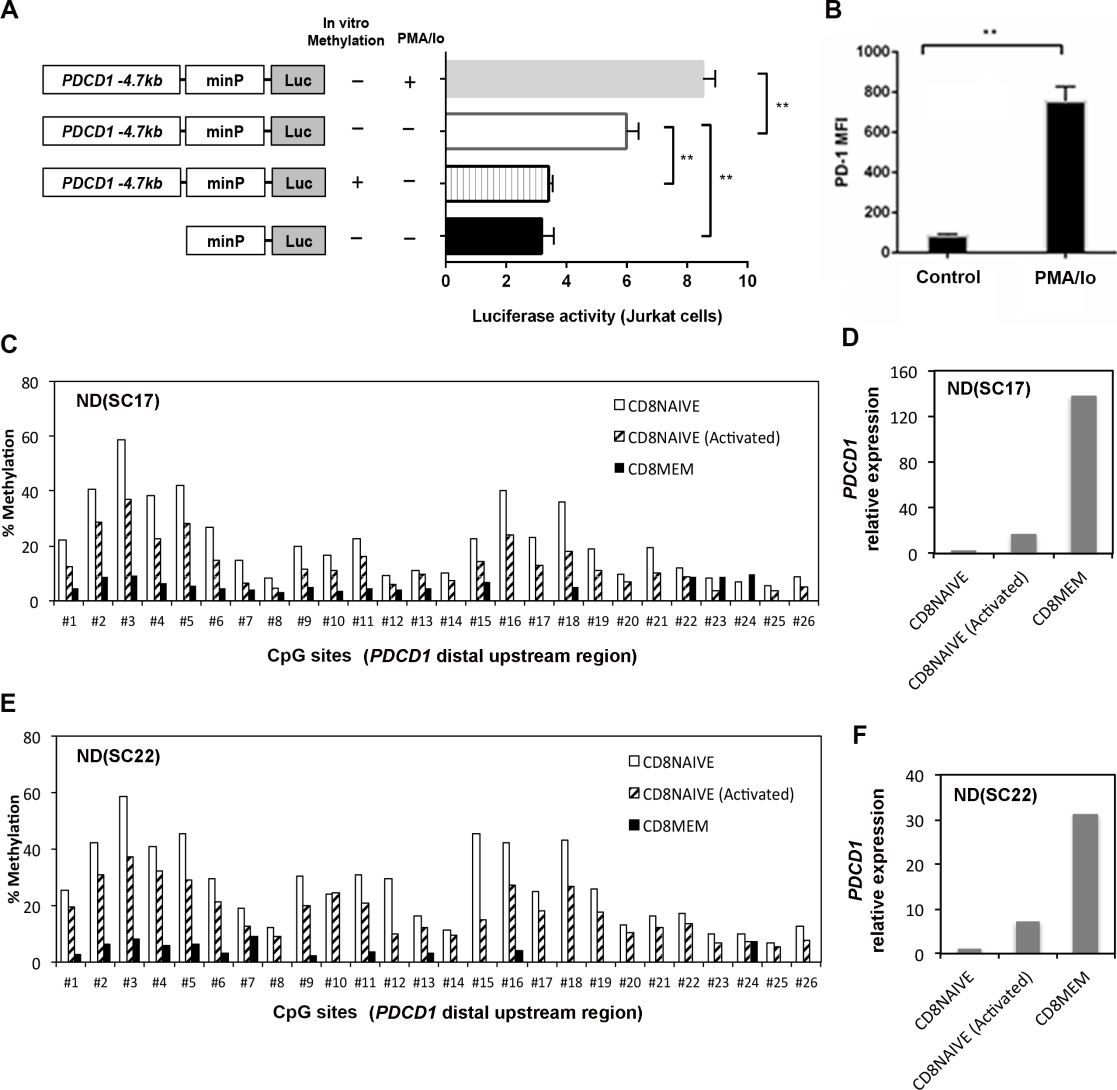
CpG methylation regulates enhancer activity of a distal upstream sequence of *PDCD1* (**A**) Luciferase reporter assays: a 677 *bp PDCD1* distal upstream DNA fragment was cloned into luciferase reporter vector pGL4.23 with a minimal promoter. Additionally, a version of the reporter plasmid was methylated by the M.SssI enzyme *in vitro*. The methylated, unmethylated, and empty reporter plasmids were then transfected into Jurkat cells to determine enhancer-mediated transcription. Schematic diagrams of experiment designs were shown on the left. (Errors bars = S.E.M.; *p < 0.05; **p < 0.01). (**B**) Surface expression of PD-1 was upregulated in normal CD8^+^ T cells upon stimulation by PMA/ionomycin. The mean fluorescence intensity (MFI) was measured by flow cytometry. (**C** and **E**) DNA methylation level of the *PDCD1* distal upstream region was measured by bisulfite pyrosequencing in purified naïve, memory CD8^+^ T cells and activated naïve CD8^+^ T cells from two independent ND samples. The first 7 CpG sites are the same CpGs analyzed in the far right panel of Figure 3B. (**D** and **F**) qRT-PCR analysis of *PDCD1* mRNA expression in purified naïve, memory CD8^+^ T cells and activated naïve CD8^+^ T cells from two ND samples. The relative expression of *PDCD1* is calculated as fold changes using the ΔΔCt method. Naïve CD8^+^ T cells were activated by anti-CD3/anti-CD28 antibodies in the presence of IL2 for 6 days and then harvested for methylation and gene expression analysis.

Because histone H3K27 acetylation is often associated with gene enhancers, we hypothesize that the *PDCD1* distal upstream region (−4.7 kb) may act as an enhancer, and CpG methylation levels within this regulatory region may control its enhancer activity. To test this hypothesis, a 677 bp sequence (−4340 to −5016 bp) of the *PDCD1* upstream locus was cloned into the pGL4.23 promoter luciferase reporter plasmid, which harbors a minimal promoter adjacent to the cloning sites. The methylated, unmethylated, and empty reporter plasmids were transfected into Jurkat cells, and luciferase activity was determined respectively (Figure 4A). We observed that the relative luciferase activity of the unmethylated reporter construct was higher than the methylated reporter construct in Jurkat cells (*p* = 0.004). Analysis of the 677 bp sequence using PROMO (http://alggen.lsi.upc.es/cgi-bin/promo_v3/promo/) identified multiple NFAT and STAT consensus binding sites. Previously, it was demonstrated that the calcineurin-NFAT pathway is critical to phorbol 12-myristate 13-acetate and ionomycin (PMA/Io) induced PD-1 expression in CD8^+^ T cells [21]. Furthermore, the luciferase activity increased significantly upon PMA/Io stimulation in Jurkat cells (*p* = 0.005), suggesting that this 677 bp sequence may serve as an upstream enhancer in regulating PD-1 expression. PMA/ionomycin stimulation also upregulated PD-1 surface expression in CD8^+^ T cells as demonstrated by the increased median fluorescence intensity (MFI) (Figure 4B).

### Activation of primary human naïve CD8^+^ T cells causes DNA methylation changes

To further confirm whether DNA methylation in the −4.7 kb region regulates PD-1 expression in human primary T cells, we purified naïve CD8^+^ T cells (CD8^+^CD45RA^+^CCR7^+^) and memory CD8^+^ T cells (CD8^+^CD45RO^+^) and analyzed the CpG methylation level within the −4.7 kb upstream locus. We found that naïve CD8^+^ T cells have higher methylation levels in this region as compared to memory CD8^+^ T cells in two independent ND samples (Figures 4C and 4E). Furthermore, the higher methylation levels in naïve CD8^+^ T cells correlated with significantly lower *PDCD1* transcript expression as compared to memory CD8^+^ T cells. However, when naïve CD8^+^ T cells were activated by anti-CD3/anti-CD28 antibodies in the presence of IL2 for 6 days, we observed decreased methylation in the −4.7 kb upstream region (Figures 4C and 4E) in the activated naïve CD8^+^ T cells. The decreased methylation in naïve CD8^+^ T cells during activation is accompanied by up-regulation of *PDCD1* mRNA expression, though its expression is still lower than purified memory CD8^+^ T cells (Figures 4D and 4F). Since we only analyzed a single time point, we may have missed the points when *PDCD1* expression reaches to the peak during anit-CD3/anti-CD28 activation, which approximately occurs around day three of activation [17].

### Genome-wide analysis of DNA methylation identifies epigenetic changes in CLL CD8^+^ T cells

**Figure 5:**
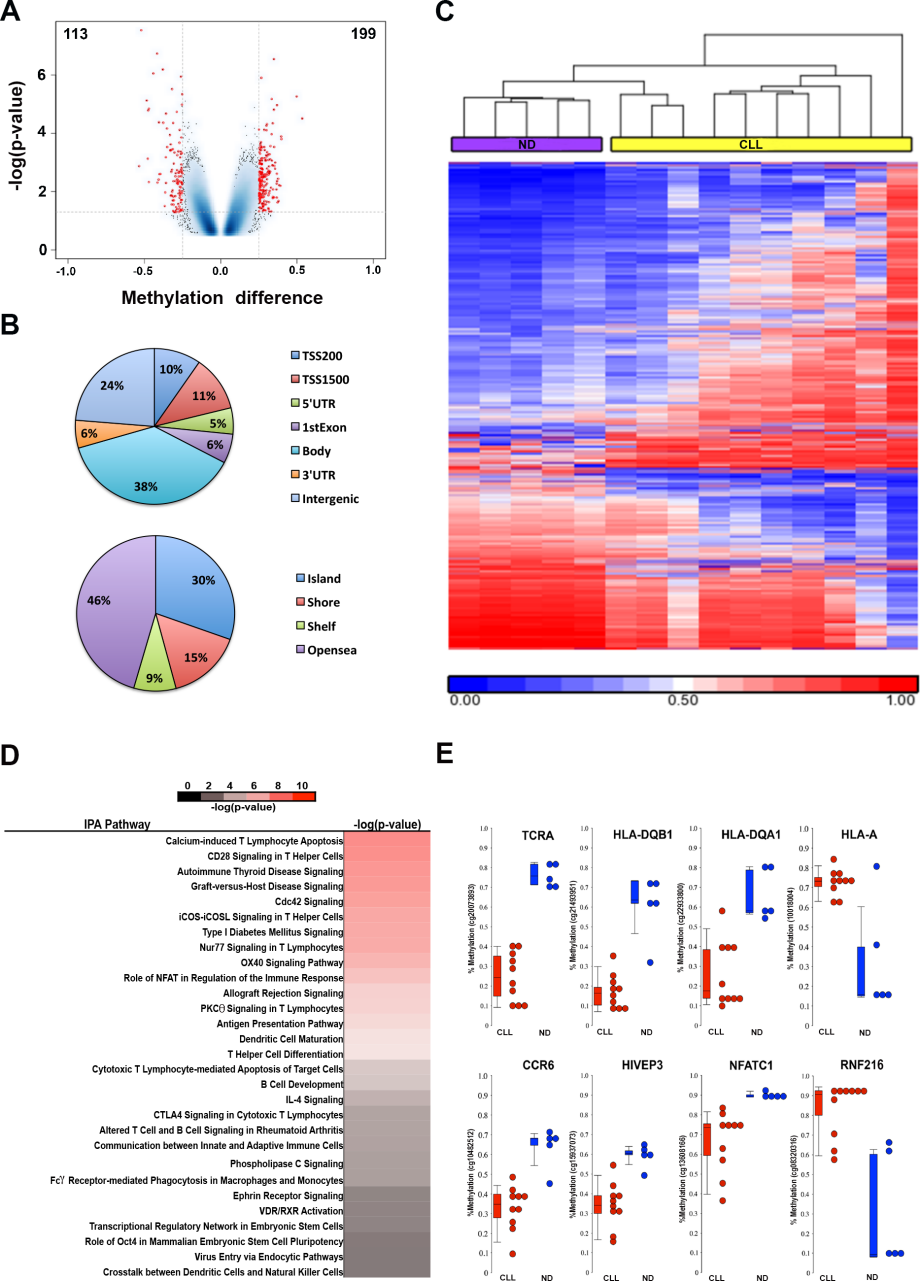
Genome-wide DNA methylation analysis identifies differentially methylated CpG sites in CD8^+^ T cells from CLL patient (*n* = 10) and ND (*n* = 5) samples (**A**) Volcano plot illustrates the 312 differentially methylated CpGs (DMCs) between CD8^+^ T cells from CLL and ND samples with 199 hypermethylated and 113 hypomethylated CpG sites that are associated with 206 genes. (**B**) Pie charts show distribution of DMCs among different annotation categories. (**C**) Heatmap shows a supervised cluster analysis of the 312 DMCs between CD8^+^T cells from CLL and ND samples. (**D**) Ingenuity Pathway Analysis revealed the enrichment of immune related pathways involved by 206 differentially methylated genes.(**E**) Representative DMCs associated with 8 genes related to immune function.

Encouraged by the discovery of DNA hypomethylation in the upstream enhancer region of *PDCD1*, we hypothesize that phenotypic changes of CD8^+^ T cells in CLL may involve epigenetic alterations in additional genes and pathways. To test this hypothesis, we performed a genome-wide DNA methylation analysis in CD8^+^ T cells from ND (*n* = 5) and CLL patients (*n* = 10) using the Illumina 450K methylation array platform. Using Student's *t*-test, a total of 312 CpG sites associated with 206 genes were deemed differentially methylated between CLL and control CD8^+^ T cells, of which 199 were hypermethylated and 113 were hypomethylated (*p* < 0.05; an average methylation difference > 0.25) (Figure 5A and Supplementary Table S1). The 312 CpG sites are associated with all annotation categories; 32% of the 312 CpG sites are located in the 5′ regulatory regions (including promoter, 5′-UTR and 1^st^ Exon), while a majority of sites are located in the gene body, 3′-UTR and intergenic regions (Figure 5B). Supervised hierarchical cluster analysis demonstrated separation between CLL and ND CD8^+^ T-cell groups based on the methylation profiles (Figure 5C). Analysis of the 206 differentially methylated genes using the Ingenuity Pathway Analysis (IPA) software revealed enrichment of largely immune related pathways (Figure 5D). Figure 5E illustrates eight representative differentially methylated CpG sites and their associated genes when comparing CLL and ND CD8^+^ T cells, respectively. These eight genes include six hypomethylated genes (*TCRA*, *HLA-DQB1*, *HLA-DQA1*, *CCR6*, *HIVEP3*, and *NFATC1*) and two hypermethylated genes (*HLA-A* and *RNF216*), all of which play important roles in regulation of T-cell functions.

**Figure 6:**
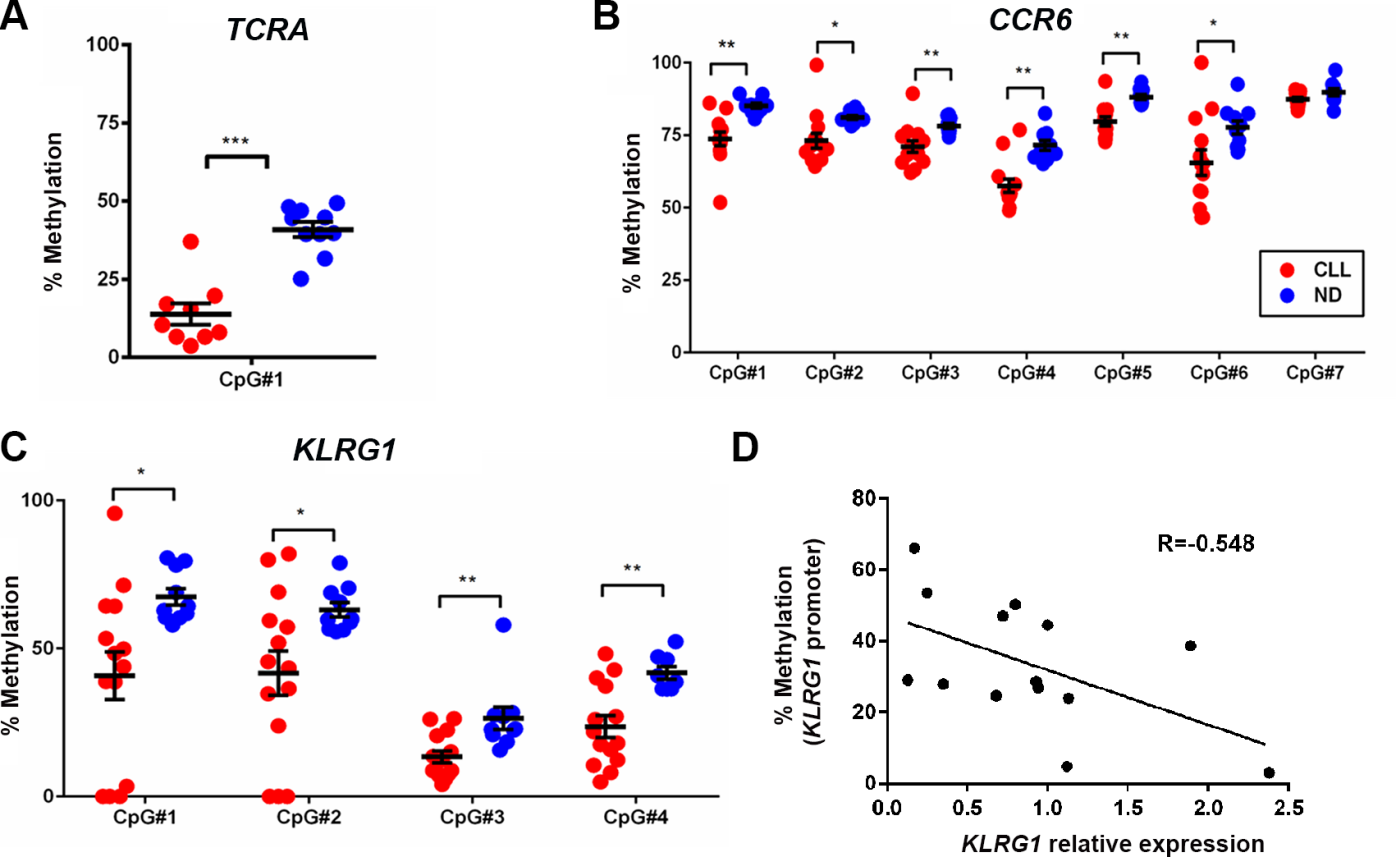
Bisulfite pyrosequencing confirmed the decrease in methylation levels of CpG sites associated with *TCRA*, *CCR6*, and *KLRG1* in CD8^+^ T cells from CLL patients as compared to healthy donors (**A**, **B**, and **C**) A representative set of DMCs from the *TCRA*, *CCR6*, and *KLRG1* genes identified by 450 K methylation-array analysis was validated by pyrosequencing. Red dots denote CD8^+^ T cells from CLL (*n* = 13–14) and blue dots represent ND samples (*n* = 10). (**D**) Quantitative RT-PCR analysis revealed inverse correlation between *KLRG1* mRNA and promoter methylation in CD8^+^ T cells from CLL patients.

To validate the array results, we performed bisulfite pyrosquencing analysis on three candidate loci associated with *TCRA*, *CCR6* and *KLRG1* from the array cohort as well as from additional primary CLL samples. The pyrosequencing results not only confirmed the hypomethylation at the CpG sites analyzed by the Illumina 450K array, but also demonstrated that the neighboring CpG sites were consistently hypomethylated in CLL compared to ND CD8^+^ T cells (Figures 6A–6C). To determine whether the differential methylation in the *CCR6* and *KLRG1* promoters can affect gene expression, we performed quantitative RT-PCR using the same set of samples and observed that average CpG methylation inversely correlated with relative gene expression of *KLRG1* (*r* = − 0.548, *p* = 0.043) (Figure 6D), but not *CCR6* (data not shown), suggesting that *KLRG1* and *CCR6* may be regulated by different epigenetic mechanisms.

## DISCUSSION

It is well established that immune dysfunction is a critical factor in CLL development. Nevertheless, the exact mechanism leading to T-cell dysfunction in CLL is not well defined. In this study, we first conducted a retrospective analysis of the number of CD4^+^ and CD8^+^ T cells in a large cohort of Chinese CLL patients. Similar to previous results derived from both US and European CLL cohorts [18, 19], we found that nearly a quarter of CLL patients exhibit inverted CD4/CD8 ratios and are associated with poor TTFT and OS. Further analysis indicates that PD-1, an indicator of T-cell exhaustion, is upregulated in the CD8^+^ T cells of CLL patients when compared to normal controls, which is in agreement with a previous report [20]. PD-1 is an inhibitory cell surface receptor required for the induction and maintenance of T-cell tolerance [22], and ligation of PD-1 on T cells by PD-L1 or PD-L2 inhibits PI3K activity and AKT activation, ultimately reducing the proliferative capability of T cells [23]. Recent evidence suggests that the PD-1/PD-L1 axis may also contribute to T-cell exhaustion in CLL [24].

Previous investigation in acute and chronic viral infection models have shown that while acute viral infection induces PD-1 expression in antigen-specific CD8^+^ T cells through transient DNA demethylation of *Pdcd1* locus, chronic exposure to viral antigens resulted in exhausted CD8^+^ T cells and complete demethylation of *Pdcd1* regulatory sequences [17]. In our study, we identified CLL-specific hypomethylation in the −4.7 kb regulatory region in CLL CD8^+^ T cells. This region contains multiple NFAT and STAT binding sites and may serve as an enhancer to *PDCD1*. Experiments using luciferase reporter plasmids revealed that DNA methylation has the potential to interfere with the enhancer activity of the −4.7 kb region. Likewise, after activating naïve CD8^+^ T cells with anti-CD3/anti-CD28 antibodies in the presence of IL2 for 6 days, we observed an increase in *PDCD1* mRNA expression and demethylation in the −4.7 kb upstream locus in activated naïve CD8^+^ T cells. Among the three regions examined, the average methylation level of the −4.7 kb regulatory region inversely correlated with *PDCD1* mRNA expression, thus further emphasizing the potential role of enhancer-mediated regulation of *PDCD1* expression. In addition, the average methylation level of the −4.7 kb region was higher in normal naïve CD8^+^ T cells than memory CD8^+^ T cells, suggesting that activation and differentiation of naïve CD8^+^ T cells are accompanied with demethylation within this distal locus. Therefore, we have identified a new *PDCD1* regulatory region with candidate distal enhancer activity in human CD8^+^ T cells. This *cis*-acting element may interact with *PDCD1* promoter elements to exert long-range control of *PDCD1* expression in human CD8^+^ T cells [25].

Currently, the mechanisms involved in demethylation of the *PDCD1* locus are still unclear; it is possible that the methylation alterations we observed at the −4.7 kb regulatory region are secondary to changes in transcription factor binding; in other words, they may not be causative. The decreased methylation levels of the −4.7 kb region in CLL CD8^+^ T cells compared to ND samples may also be caused by preferential expansion of CD8^+^ effector memory T cells with significantly lower methylation levels in this region. However, to obtain more precise results, further purification of CLL neoantigen-specific T cells may be required since dynamic DNA methylation changes in the *PDCD1* locus have been observed in viral antigen-specific CD8^+^ T cells [17]. Nevertheless, the hypomethylation of the −4.7 kb region observed in CLL CD8^+^ T cells is statistically significant and can be consistently observed in CLL patient samples. Our results suggest that PD-1 upregulation and corresponding T-cell exhaustion in CLL may be caused by chronic exposure to tumor antigens, and this upregulation is mediated through DNA demethylation similarly observed in chronic viral infections.

Several studies using global gene expression analysis demonstrated that T cells from patients with CLL show a number of differentially expressed genes when compared to age-matched healthy donor T cells [4]. However, gene expression signatures alone cannot fully explain the underlying mechanisms. Within our study, we performed genome-wide DNA methylation profiling on CD8^+^ T cells from CLL patients and healthy controls in order to identify DNA methylation changes in CD8^+^ T cells from patients with CLL. We observed recurrent methylation changes in additional immune related genes including *TCRA*, *HLA-DQB1*, *HLA-DQA1*, *CCR6*, *HIVEP3*, *NFATC1*, and *KLRG1*. Using quantitative pyrosequencing in a cohort of CLL CD8^+^ T-cell samples, we further confirmed the aberrant methylation changes in *CCR6* and *KLRG1*. Interestingly, we found that, similar to *PDCD1*, DNA hypomethylation of the *KLRG1* locus also inversely correlated with mRNA expression within the CD8^+^ T cells of CLL patients. Pathway analysis using the Ingenuity Pathway Analysis software package revealed enrichment of immune related pathways. Of particular note, NFAT signaling is involved in several top pathways, which is consistent with recent findings that the transcription factor NFAT promotes exhaustion of activated CD8^+^ T cells [26]. One limitation of our genome-wide DNA methylation study in CLL CD8^+^ T cells is the small sample size, which gives us limited statistical power. However, we included additional criteria for selecting differentially methylated genes and were able to validate several candidate genes using an independent approach. Nevertheless, future large-scale studies are needed to confirm the discovery made in this pilot study.

The chemokine receptor CCR6 is a receptor for the inflammatory chemokine CCL20. CCR6 is widely expressed on human leukocytes including circulating memory CD8^+^ T cells, regulatory T cells, and immature dendritic cells (DCs) [27, 28]. A study showed that CCR6^+^CD8^+^ T cells are an effector memory phenotype T-cell subset and have the ability to migrate in response to CCL20 [28]. The DNA demethylation in the *CCR6* promoter DMR locus is functionally important for maintaining long-term stability of CCR6 expression in memory T cells [29]. It seems that demethylation of the *CCR6* promoter in CLL CD8^+^ T cells did not correlate with *CCR6* mRNA upregulation, suggesting the involvement of other mechanisms in the active transcription of *CCR6* in CLL CD8^+^ T cells.

Consistent with our DNA methylation data, it was previously reported that expanded CD8^+^ T cells of murine and human CLL are driven into a senescent KLRG1^+^ effector memory phenotype [12]. KLRG1 is one of the C-type lectin natural killer (NK) cell inhibitory receptors that contain an immunoreceptor tyrosine-based inhibitory motif (ITIM) [30]. KLRG1 is selectively expressed in effector and memory CD8^+^ T cells [31, 32]. Particularly, KLRG1 identifies human CD8^+^ T cells that secrete cytokines but fail to proliferate upon stimulation, a phenomenon that frequently exists among CD8^+^ T cells in CLL patients [11]. It is well documented that KLRG1 is upregulated on CD8^+^ T cells during both viral and parasitic infections, and its expression is maintained on CD8^+^ T cells post viral infection [33]. Therefore, our data suggests that KLRG1 upregulation in CD8+ T cells from CLL patients may also involve epigenetic reprograming through similar mechanisms as PD-1.

Several proteins such as fibromodulin, CD229, MDM2, and CD23 have been recognized as tumor-associated antigens in CLL [34–37]. Recent studies also identified personal tumor-specific neoantigens in CLL, suggesting the potential for chronic antigen stimulation in CLL patients [38]. Our results revealed for the first time an underlying epigenetic change in CLL CD8^+^ T cells that is potentially driven by long-term exposure to tumor-related antigens. However, further studies in the subset of tumor antigen-specific CD8^+^ T cells are needed to reveal the precise mechanisms of action. Taken together, the data presented supports the presence of epigenetic reprogramming of immune related genes within CD8^+^ T cells of CLL patients. Our study provides new evidence that epigenetic profiling of antigen-specific CD8^+^ T cells may provide novel parameters when assessing T-cell memory quality and may help improve the effectiveness of future immunotherapy strategies in CLL. Given the importance of the PD-1/PD-L1 pathway in regulating T cell activation and tolerance, therapeutic blockade of the PD-1/PD-L1 axis may improve the outcome of CLL patients affected by immune dysregulation.

## MATERIALS AND METHODS

### Blood samples from healthy donors and CLL patients

Blood samples were collected from CLL patients at Georgia Cancer Center at Augusta University, Augusta, Georgia and the First Affiliated hospital of Nanjing Medical University, Nanjing, Jiangsu, China. The population for the retrospective study consisted of 234 untreated CLL patients between June 2006 and July 2014 from clinics at the First Affiliated hospital of Nanjing Medical University (age range: 20–86 years). A control group of 10 age-matched normal donors were also recruited (age range: 20–75years). All patients and donors provided informed consent based upon the guidelines of the local Institutional Review Boards at each institution. Data collected at diagnosis included: age, gender, and Binet stages. A number of other prognostic markers were also analyzed for a large subset of these patients including IGHV mutation, ZAP70 and CD38 expression, TP53 mutation, and cytogenetic status. Other clinical data analyzed included peripheral blood CD4^+^ and CD8^+^ T cells percentage (*n* = 234), and absolute counts of the cells at the time of diagnosis or within 6 months after diagnosis (*n* = 209).

### Purification of T cell subsets

Peripheral blood mononuclear cells (PBMCs) were isolated using a Ficoll-Hypaque density gradient and either used immediately or stored in liquid nitrogen. CD8^+^ T cells from normal PBMCs were negatively selected using EasySep CD8^+^ T-cell isolation kit (StemCell Technologies). To isolate CD8^+^ T cells from CLL patient samples, CD19^+^ B cells were first depleted using EasySep B-cell isolation kit (StemCell Technologies) before undergoing CD8^+^ T cells negative selection. To further enrich memory and naïve T cells from negatively selected CD8^+^ T cells, we used biotin-conjugated antibodies against CD45RO and CD244 (2B4), which are predominantly expressed by effector and memory T cells, for positive selection of memory T cells. The flow-through fraction is untouched naïve CD8^+^ T cells. Using this approach, we can obtain high purity of naïve and memory CD8^+^ T cells (greater than 90% purity).

### Cell culture and T-cell activation

The PBMCs and Jurkat cells were cultured in RPMI-1640 media supplemented with 10% fetal bovine serum (FBS) at 37°C and 5% CO_2_. Primary PBMCs were stimulated with 50 ng/mL PMA and 1 μM ionomycin. Following 72h incubation, PD-1 surface expression on CD8^+^ T cells was measured by flow cytometry. Purified naïve CD8^+^ T cells were stimulated with 5μg/mL immobilized anti-CD3 (clone OKT3, eBioscience), 2 μg/mL soluble anti-CD28 (clone CD28.2, eBioscience), and 100 ng/mL of IL2 (BioLegend) for six days. Cells were then harvested for pyrosequencing and qRT-PCR analysis.

### Monoclonal antibodies and flow cytometry

Cells were stained using fluorochrome-coupled monoclonal antibodies (mAbs). The following mAbs were used in this study. CD3-PECy7, CD3-FITC, CD4-AlexaFluor488, CD4-PerCP/Cy5.5, PD-1-BrilliantViolet421, CD8-APC CD8-PerCP/Cy5.5, CD45RO-PE, CCR7-PE, and CD45RA -AlexaFluor700 were all obtained from BD Bioscience. CD8-FITC, PD-1-PE, PD-1-APC, and CD3-APC were obtained from eBioscience. CD244-PE, CD4-PE/Cy5, CD8-PE/Cy5, CD3-FITC, CD19-FITC, CD19-AlexaFluor700 and CD19-BrilliantViolet421 were obtained from BioLegend. Flow cytometry analyses were performed on a LSRII flow cytometer (BD Biosciences) and subsequently analyzed using Flowjo, version 9.0.2 software (ThreeStar).

### Quantitative real-time PCR

Total RNA from the corresponding samples was converted into cDNA using High Capacity cDNA synthesis kit (Applied Biosytems). qRT-PCR was performed in triplicates on a CFX96 real time PCR instrument (BioRad). mRNA levels of target genes were normalized to β-Actin. The primer sequences are available upon request.

### Bisulfite pyrosequencing

To determine the methylation status of CpG sites of *PDCD1*, pyrosequencing of multiple sites across the 1^st^ intron (+209 to +589), distal promoter (−940 to −1162), and distal upstream (−4673 to −4977) regions were performed. The bisulfite pyrosequencing analysis was carried out using the PyroMark Q24 instrument, according to manufacturer's instructions (Qiagen). PyroMark Q24 software was used to analyze the program outputs by calculating the ratio of relative peaks of methylated vs. unmethylated alleles. The bisulfite PCR and sequencing primers are available upon request.

### Construction of plasmids and luciferase reporter assays

A 677 bp fragment encompassing the PD-1 distal upstream region (−4340 to −5016 bp) was amplified by PCR and then cut with HindIII/XhoI (NEB) before cloned into HindIII/XhoI sites of firefly luciferase reporter vector pGL4.23 (Promega). The constructs were verified by partial sequencing. To test the effect of CpG methylation on the reporter activity, we methylated the reporter plasmid DNA *in vitro* using CpG Methyltransferase *M.Sss*I (NEB). Methylated or unmethylated plasmids were transiently transfected into Jurkat cells using Lipofectamine LTX reagents (Invitrogen). At 24 h post transfection, Jurkat cells were either stimulated with 50 ng/mL PMA and 1 μM ionomycin or vehicle control. Lysates were collected 24 h after PMA/ionomycin addition. A Dual-Luciferases Reporter Assay System (Promega) was used to quantify luciferase activities. Firefly luciferase was normalized to the cotransfected (20:1), constitutively expressing Renilla luciferase.

### Illumina Infinium 450K methylation array analysis

The bisulfite conversion of genomic DNA was carried out using EZ DNA Methylation Gold kit (Zymo Research). 200 ng converted DNA was analyzed using Illumina Infinium 450K Methylation array according to manufacturer's suggested protocols (Illumina). The quantitative value of DNA methylation was calculated from the ratio of fluorescent signals from the methylated alleles to the sum of the signals from the methylated and unmethylated alleles, assigned via Genome Studio Methylation Software Module (Illumina). Minfi package in Bioconductor (http://bioconductor.org/packages/release/bioc/html/minfi.html) was used for quality control and normalization before statistical analysis. Student *t*-test analysis from Limma package was used to identify differentially methylated genes with statistical significance between ND and CLL CD8^+^ T cells in methylation array data. A *p*-value < 0.05 and methylation difference > 0.25 were the cut off values used to select differentially methylated CpG sites.

### Statistics

Analysis of prognostic subsets was carried out using Fisher's exact test. Nonparametric Mann-Whitney U test was used for comparison of 2 independent groups. Overall survival time was calculated from date of diagnosis and curves were constructed using the Kaplan-Meier method. Correlation coefficients were determined using the parametric linear regression analysis. The difference between experimental groups was assessed by two-tailed unpaired Student's *t*-test or one-way ANOVA with Tukey post-hoc tests. The methylation levels of CpG sites between CLL patients and ND samples were compared by multiple *t* tests. All statistical analyses were carried out using GraphPad Prism 6.0 or SPSS Statistics version 18.0 as indicated in the text at a significance level of *p < 0.05; **p < 0.01; ***p < 0.001.

## SUPPLEMENTARY MATERIALS TABLE




