# Supplementary material for: Phenotypic alteration of CD8+ T cells in chronic lymphocytic leukemia is associated with epigenetic reprogramming
Source: Oncotarget. 2016 Jun 10;7(26):40558–70. doi: 10.18632/oncotarget.9941 (PMC5130028; doi:10.18632/oncotarget.9941)
Supplement: Supplementary file 1 [file oncotarget-07-40558-s001.pdf]

Phenotypic alteration of $CD8^+$ T cells in chronic lymphocytic leukemia is associated with epigenetic reprogramming

Supplementary Materials

See Supplementary Table S1.
